# Targeted Echocardiographic Screening for Latent Rheumatic Heart Disease in Northern Uganda: Evaluating Familial Risk Following Identification of an Index Case

**DOI:** 10.1371/journal.pntd.0004727

**Published:** 2016-06-13

**Authors:** Twalib Aliku, Craig Sable, Amy Scheel, Alison Tompsett, Peter Lwabi, Emmy Okello, Robert McCarter, Marshall Summar, Andrea Beaton

**Affiliations:** 1 School of Medicine, Gulu University, Gulu, Uganda; 2 Division of Cardiology, Children’s National Health System, Washington, District of Columbia, United States of America; 3 Uganda Heart Institute, Kampala, Uganda; 4 School of Medicine, Makerere University, Kampala, Uganda; 5 Division of Biostatistics and Informatics, Children’s National Health System, Washington, District of Columbia, United States of America; 6 Division of Genetics and Metabolism, Children’s National Health System, Washington, District of Columbia, United States of America; University of California San Diego School of Medicine, UNITED STATES

## Abstract

**Background:**

Echocardiographic screening for detection of latent RHD has shown potential as a strategy to decrease the burden of disease. However, further research is needed to determine optimal implementation strategies. RHD results from a complex interplay between environment and host susceptibility. Family members share both and relatives of children with latent RHD may represent a high-risk group. The objective of this study was to use echocardiographic family screening to determine the relative risk of RHD among first-degree relatives of children with latent RHD compared to the risk in first-degree relatives of healthy peers.

**Methodology/Principal Findings:**

Previous school-based screening data were used to identify RHD positive children and RHD negative peers. All first-degree relatives ≥ 5 years were invited for echocardiography screening (2012 World Heart Federation Criteria). Sixty RHD positive cases (30 borderline/30 definite RHD) and 67 RHD negative cases were recruited. A total of 455/667 (68%) family members were screened. Definite RHD was more common in childhood siblings of RHD positive compared to RHD negative (p = 0.05). Children with any RHD were 4.5 times as likely to have a sibling with definite RHD, a risk that increased to 5.6 times when considering only cases with definite RHD. Mothers of RHD positive and RHD negative cases had an unexpectedly high rate of latent RHD (9.3%).

**Conclusions/Significance:**

Siblings of RHD positive cases with RHD are more likely to have definite RHD and the relative risk is highest if the index case has definite RHD. Future screening programs should consider implementation of sibling screening following detection of an RHD positive child. Larger screening studies of adults are needed, as data on prevalence of latent RHD outside of childhood are sparse. Future studies should prioritize implementation research to answer questions of how RHD screening can best be integrated into existing healthcare structures, ensuring practical and sustainable screening programs.

## Author Summary

Rheumatic heart disease (RHD) affects at least 33 million people, most of who live in low-resource environments. RHD is a cumulative process and there exists a latent period between early valve damage and presentation with symptoms. Echocardiographic screening (ultrasound of the heart) has proven highly sensitive for latent RHD detection, but implementation research is needed to effectively develop sustainable public health strategies. Critical to this research is determining whom to screen. As family members have both a shared environment and shared genetic susceptibility, they may represent a high-risk group that could be targeted once a case of RHD is identified. We conducted an echocardiographic family screening study to determine the risk of RHD in families with and without an RHD positive child and found that siblings of children with latent RHD are more likely to have latent RHD themselves. Our data suggest that siblings may represent a particularly high-risk group that could be targeted for echocardiographic screening. Future studies are needed to answer questions of how RHD screening can best be integrated into existing healthcare structures, ensuring practical and sustainable RHD screening programs.

## Introduction

Rheumatic heart disease (RHD), the long-term consequence of acute rheumatic fever (ARF), is the result of a complex interplay between host and environment. Endemic areas are consistently marked by poverty, poor sanitation, and limited access to primary healthcare [1]. These factors increase the incidence of group A β-hemolytic streptococcal (GAS) carriage, infection, and transmission. Repeated, untreated GAS infections create the substrate for development of ARF, a systemic immune system over-reaction that results, for many, in RHD [2].

However, environmental exposure is only one component of RHD susceptibility. Even in the presence of endemic GAS and poor primary prevention (penicillin for acute streptococcal pharyngitis) not all children are equally at risk. ARF follows only 3–6% of cases of GAS and only 40% of children with ARF develop chronic RHD [3]. Historically, RHD was noted to cluster in families, and a meta-analysis of twin studies showed a pooled concordance risk for ARF of 44% in monozygotic twins and 12% in dizygotic twins, giving an estimated heritability of 60%[4]. The majority of these data were captured from observational studies of ARF, and pre-dated routine echocardiography [5].

In many low-resource settings today, presentation with ARF has become rare even as echocardiographic screening of school-aged children has revealed a large burden of latent RHD (RHD apparent on echocardiography that has not previously come to clinical attention). Given what is known about genetic susceptibility and a shared environment, it is reasonable to assume that family members of children with latent RHD may themselves be at greater risk of latent RHD. However contemporary echocardiographic screening of families living in RHD endemic areas has not been reported. The objective of this study was to use echocardiographic family screening to determine the relative risk of RHD among first-degree relatives of children with latent RHD compared to the risk in first-degree relatives of healthy peers.

## Materials and Methods

### General Design

We utilized a cross-sectional family design to compare the risk of RHD among first-degree family members of primary school children previously identified with latent RHD compared to the first-degree family members of age/gender matched children with normal echocardiograms. The study occurred over a 3-month period from February-April, 2015. Informed consent was obtained from all participants at least 18 years of age, and informed assent and parental permission was obtained for those between 5–17 years. Approval for this study was granted from the Institutional Review Boards at Children’s National Health System, Washington DC, Makerere University School of Medicine, Kampala, Uganda, and the Ugandan National Council of Science and Technology.

### Study Population

RHD positive index cases included children with borderline or definite RHD (2012 WHF criteria), identified through previous echocardiographic school screening programs in the Gulu District of Northern Uganda in 2014.[6] These children are followed clinically at the Gulu Regional Referral Hospital, Gulu, Uganda. RHD negative index cases were recruited from screen-negative peers who were similar in age and gender and attending the same schools (reflecting the same general socioeconomic status). All RHD positive and RHD negative index children underwent repeated echocardiographic evaluation at time of study enrollment to ensure their RHD status had not changed since first screen in 2014.

The parents/guardians of RHD positive and RHD negative index children were approached to invite them, and all first-degree relatives (≥5 years of age) in the family, to undergo echocardiographic screening to evaluate for the presence of latent RHD. Children without at least one parent alive/available were excluded.

### Clinical Data

Following family recruitment, a list of all first-degree family members (at least 5 years of age)–living or deceased was captured. For living family members, age, gender, and known history of ARF/RHD were recorded. For family members who were deceased, attempts were made to understand the cause of death. For those family members who were alive but unavailable for screening, the reason for absence was recorded.

### Echocardiographic Protocol

**Fig 1:**
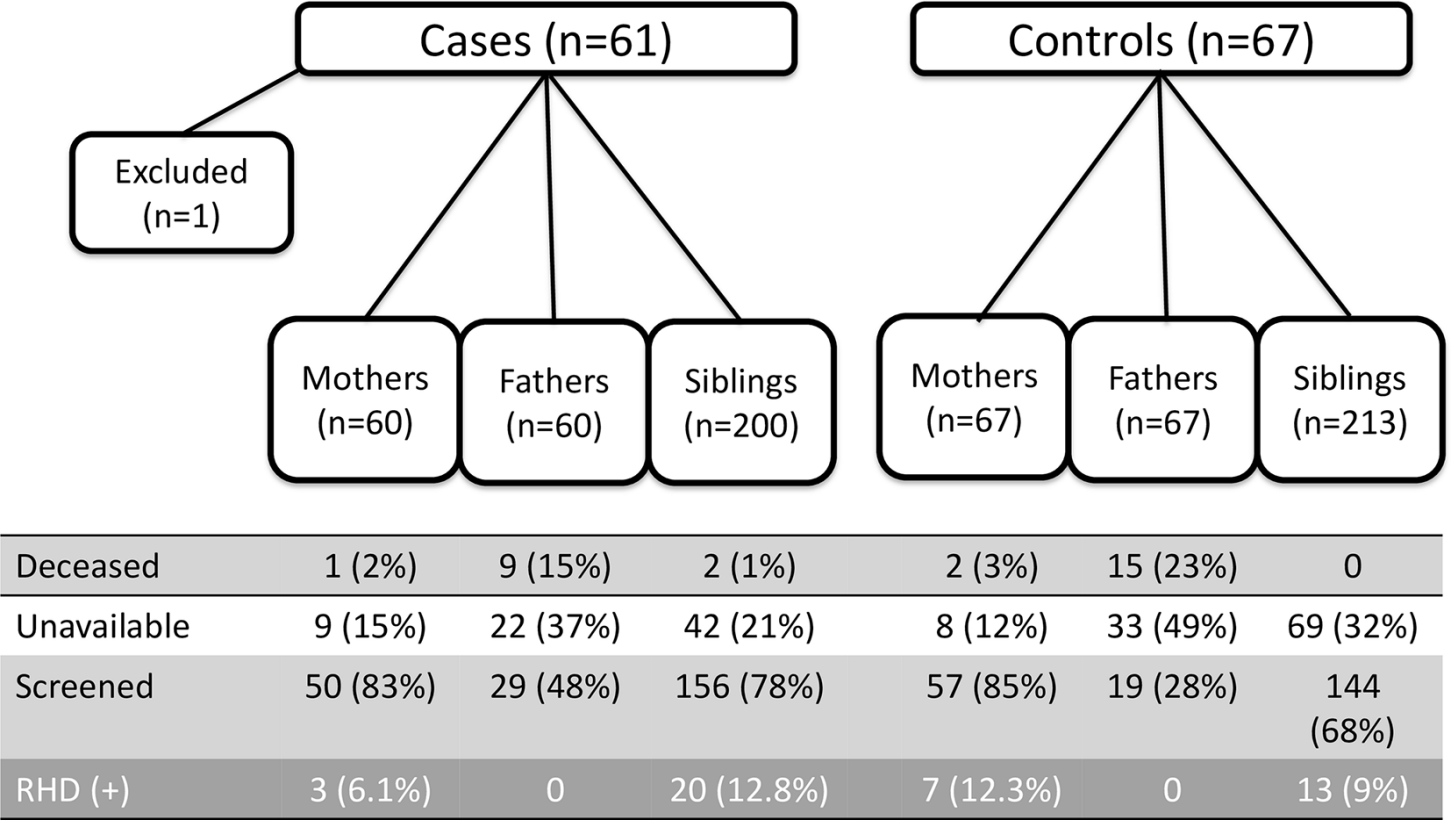
First-degree family members of RHD positive and RHD negative index cases according to status.

Each participating family member underwent a focused transthoracic echocardiogram performed by a pediatric cardiologist (TA) with expertise in RHD. A standard acquisition protocol in the parasternal long, parasternal short, and apical 4- and 5-chamber views focused on assessment of the mitral and aortic valves. Additional views were obtained when needed. All images were obtained using fully functional standard portable echocardiographic equipment (GE, VIVID Q, Milwaukee, WI) (Fig 1). Studies were transferred through a secure telemedicine system to PACS (Philips Xcelera, Best, Netherlands) for offline review. Three reviewers (AB, CS, AT), blinded to the RHD/- status of the index child reviewed studies and classified them according to the 2012 WHF criteria (S1 Table) (normal, borderline RHD, definite RHD, or other for subjects ≤20 years of age and normal, definite RHD, or other for subjects >20 years of age)[7]. All positive studies were confirmed by a second reviewer and, in cases of disagreement, a third reviewer determined the final classification.

### Statistical Analysis

Demographic information is presented by number and percentage, and where applicable with standard deviation. Continuous variables were compared using Student’s t-test. Fisher’s exact tests were used to evaluate the exact probability under the null hypothesis of observing results as or more extreme based on comparisons of differences in categorical variables between study groups. Poisson Regression was used to estimate the average risk and relative risk of RHD positivity in first-degree family members of RHD positive and RHD negative index children. The method of Poisson Regression was chosen because it appropriately handles outcomes based on counts, including low frequency counts, that generally do not meet criteria for tests requiring the data to meet the parametric assumption and appropriately accounts for the natural clustering of family data. Prevalence rates of RHD among mothers vs. fathers vs. siblings are presented as percentages and compared using model z-statistics. Relative risk was also presented according to the presence of borderline vs. definite RHD in index children. Agreement between reviewers was calculated with the Kappa statistic. Greatest emphasis was placed on results that achieved statistical significance at the p<0.05 level, but substantive differences that achieved borderline significance were also described.

## Results

The index group consisted of 61 RHD positive children (30 with definite RHD and 31 with borderline RHD) and 67 RHD negative children (Table 1), generating a complete list of 320 (5.3/child) and 347 (5.2/child) first-degree family members of RHD positive and RHD negative index subjects, respectively, p = 0.22. During enrollment, 1 child (borderline RHD) was excluded from participation when no biological parent could attend screening–leaving 60 RHD positive index cases and 67 RHD negative index cases.

**Table 1:** Demographics of Index Cases.

Index Cases	RHD positive* (n = 60)	RHD negative (n = 67)	p-value
Age (mean, ± SD)	12.5 (2.4)	12.4 (2.4)	1.00
Gender (n, % Female)	60% (n = 36)	59.7% (n = 40)	0.29
# First Degree Relatives (n, mean)	320 (5.3)	347 (5.2)	1.00

* Not including 1 case of borderline RHD excluded for no biological parents.

Of the 667 identified first-degree relatives, 455 (68.2%) attended screening including 107 mothers (83.5%), 48 fathers (37.8%), and 300 siblings (72.6%) (Table 2). Fathers (24/127, 19%) were more likely to be deceased than mothers (3/127, 2.4%, p<0.01), with the reasons for paternal death including HIV/AIDS (6), accidental trauma (5), other illness (5), the LRA conflict (3), and other/unknown (5). Fathers who were alive were also less likely to be available for screening (55/103, 53% unavailable) than mothers (17/124, 14% unavailable, p<0.01). Siblings of RHD negative cases were less available to participate in screening (p = 0.03). A breakdown of reasons for all family members who were alive but unavailable and those who are deceased are listed (Table 3). No absent family members were reported to have cardiovascular symptoms and no causes of death were known to be attributable to cardiac disease.

**Table 2:** Demographics of First-Degree Relatives.

Mothers	RHD positive	RHD negative	p-value
	(n = 60)	(n = 67)	
Deceased	1 (2%)	2 (3%)	1.00
Unavailable	9 (15%)	8 (12%)	0.79
Screened*	50 (83%)	57 (85%)	0.81
Age (mean, ± SD)	36.6 (5.7)	35.8 (7.1)	0.54
History of ARF/RHD	0	0	n/a
RHD Positive	3 (6.1%)	7 (12.3%)	0.33
Fathers	Cases (n = 60)	Controls (n = 67)	
Deceased	9 (15%)	15 (23%)	0.37
Unavailable	22 (37%)	33 (49%)	0.05
Screened*	29 (48%)	19 (28%)	0.03
Age (mean, ± SD)	44.5 (7.1)	43.5 (7.6)	0.8
History of ARF/RHD	0	0	n/a
RHD Positive	0	0	n/a
Siblings	Cases (n = 200)	Controls (n = 213)	
Deceased	2 (1%)	0	0.24
Unavailable	42 (21%)	69 (32%)	0.01
Screened*	156 (78%)	144 (68%)	0.02
Age (mean, ± SD)	12.1 (4.9)	11.0 (4.3)	0.3
History of ARF/RHD	2 (1.3%)	0	n/a
RHD Positive (Borderline**+ Definite)	20 (12.8%)	13 (9%)	0.36
Borderline RHD**	12 (7.7%)	11 (8.1%)	1.00
Definite RHD	8 (5.6%)	2 (1.5%)	0.11

*Details only for first-degree relatives who were screened

**Only in siblings ≤18 years

ARF: Acute Rheumatic Fever

RHD: Rheumatic Heart Disease

**Table 3:** Breakdown of Reasons for Unavailability or Death.

Death						
	HIV/AIDS	Accident	LRA Conflict	Other Illness	Other/Unknown	Total
Father	6	5	3	5	5	24
Mother				2	1	3
Sibling				1	1	2
Alive but Unavailable						
	Distance (lives/works far from home)	Divorced/Not in Contact with Family	Could not get off work	Boarding School/School in another city	Other/Unknown	Total
Father	20	7	22	0	6	55
Mother	0	4	6	0	7	17
Sibling	9	0	7	56	39	111

LRA: Lord’s Resistance Army

**Fig 2:**
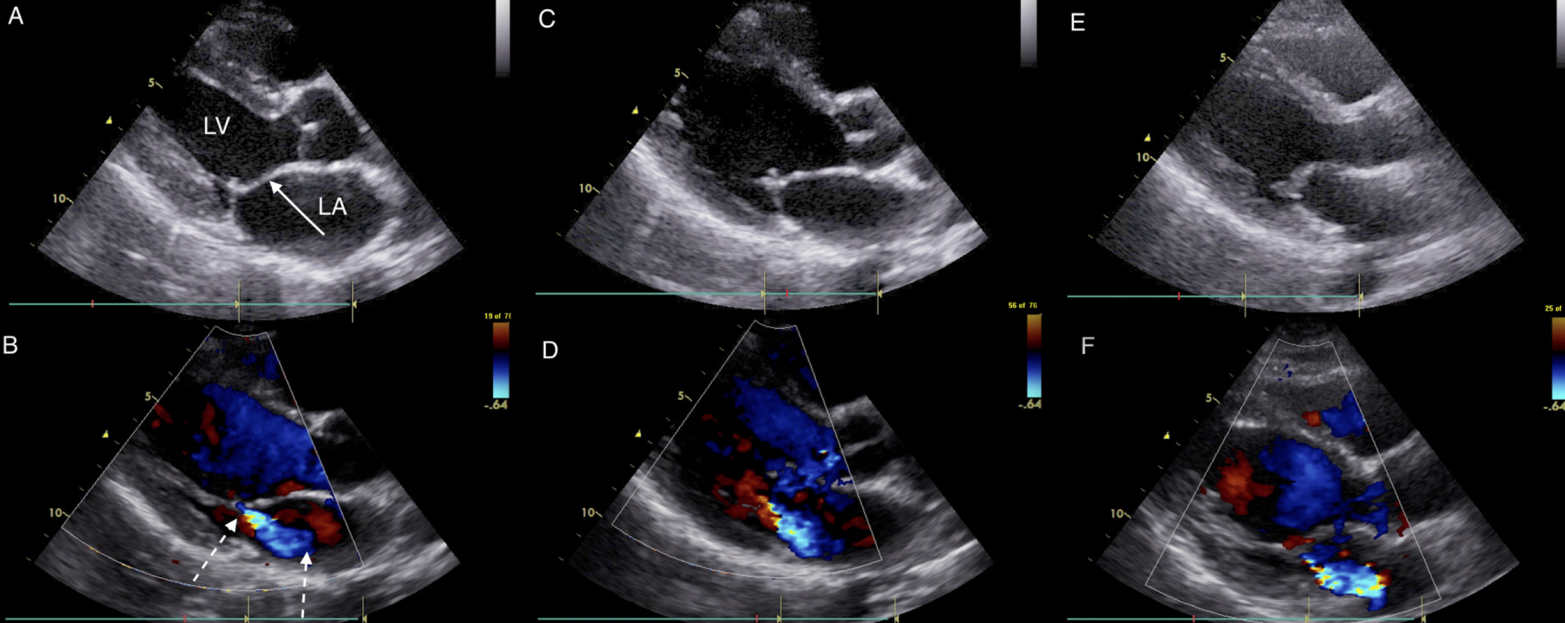
Images from a family with 3 RHD positive members: Index Case, Sibling and Mother. Parasternal long axis echocardiographic still frames in early systole in black and white (panels A, C, and E) and color Doppler (panels B, D, and F) of RHD positive index case (panels A and B), sibling (panels C and D) and mother panels (E and F). The black and white panels all show a thickened anterior mitral valve leaflet (solid arrow) and prolapse (excessive tip motion) of tip of anterior mitral valve leaflet. The mother has the most extreme thickening of the valve along with thickened chordae and immobile valve motion (not seen on these frames). The color Doppler panels show similar mitral regurgitation jets (dotted arrows) measuring approximately 3 cm in each subject. Both RHD positive index case and sibling also had aortic insufficiency jet measuring > 1 cm (not shown). Abbreviations: LA–left atrium, LV–left ventricle.

The prevalence of all latent RHD was similar in first-degree relatives of RHD positive and RHD negative cases 9.8% vs. 9.0% (23/235 screened vs. 20/220 screened, p = 0.87). Similarly there was no difference between prevalence of definite latent RHD between groups (Cases: 11/235, 4.3% vs. Controls: 9/220, 4.1%, p = 1.00). Definite RHD was more likely to be found in mothers, with 9.3% (10/107 screened) having echocardiographic evidence of definite RHD, compared to fathers 0% (0/48 screened, p = 0.03), and siblings 3.3% (10/300 screened, p = 0.02). Borderline RHD, a category reserved only for those ≤20 years of age, was similar prevalence between siblings of RHD positive vs. RHD negative (7.7% vs. 8.1%, p = 1.00). However, definite RHD was more common among siblings of RHD positive cases (5.2% vs. 1.4%, p = 0.11), but only reached borderline significance. There were 7 families (4 cases, 3 controls) where 2 or more first-degree relatives were found to be RHD positive (Fig 2).

There was no increased familial, or sibling risk of RHD in the first-degree relatives of RHD positive cases (borderline & definite RHD) vs. RHD negative cases. However, RHD positive cases had a 4.5 times greater chance of having a sibling with definite RHD (p = 0.05) and this risk increased to 5.6 times greater chance if you limited the comparison to RHD positive cases with definite RHD (n = 30, p = 0.03) (Table 4).

**Table 4:** Relative Risk of Rheumatic Heart Disease.

	Relative Risk	p-value
If any RHD–risk of having another affected first-degree relative (23/235 vs. 20/220)	1.05 (0.6–1.8)	0.85
If any RHD–risk of having a family member with definite RHD (11/255 vs. 9/232)	1.2 (0.54–2.8)	0.61
If any RHD–risk of having a sibling with definite RHD (8/60 vs. 2/67)	4.5 (1–20.2)	0.052
If definite RHD–risk of having a sibling with definite RHD (5/30 vs. 2/67)	5.6 (1.1–27.2)	0.033

RHD: Rheumatic Heart Disease

There was 97% agreement between reviewers 1 and 2 (κ = 0.86, 95% CI 0.78–0.93), with 13 cases of non-agreement adjudicated by the third reviewer. All cases of non-agreement were between the diagnoses of “borderline RHD” or “normal” with 100% agreement on the diagnosis of definite RHD.

## Discussion

This is the first study to assess the utility of echocardiography screening of first-degree relatives of children with latent RHD. Siblings of RHD positive cases with any RHD are more likely to have definite RHD and the relative risk goes from 4.5 to 5.6 if the index case has definite RHD. Additionally, we found that nearly 10% of mothers had latent RHD by echocardiography while no fathers were positive. Unlike our sibling results, the likelihood of a mother being positive for RHD has no association with a positive index case.

Echocardiographic screening has shown potential as a public health strategy to decrease the global burden of RHD. Population studies, mostly involving schoolchildren, have revealed a weighted pooled prevalence of 1.3% of children living in endemic areas show evidence of latent RHD [8]. Early detection of these children provides the opportunity for secondary prophylaxis, monthly penicillin injections that prevent recurrent streptococcal infections, rheumatic fever, and further valve damage. This is of particular importance in areas such as sub-Saharan Africa where RHD remains endemic, ARF rarely comes to clinical attention [9], and RHD patients most commonly present late, with advanced disease and resulting complications [10].

Optimal implementation strategies, the who, when, in what setting, and how often to screen, have received little study to date, yet these details are critical to developing cost-effective and sustainable screening programs. Our study suggests that siblings of children identified with latent RHD are a high-risk group, and should be prioritized for screening. Siblings of index controls showed a 1.4% prevalence of definite RHD, which is comparable to previously published data from the pediatric population in Gulu, Uganda [6]. In contrast, siblings of RHD positive cases were found to have a 5.2% prevalence of latent, but definite RHD–and to be at 4.5–5.6 times risk, depending if you included index cases with borderline and definite RHD or only those with definite RHD respectively. In a resource-constrained setting, identification of this increased risk could translate into strategic targeting of siblings for single or repeated echocardiographic screening and for education on primary prevention. Strategies such as this would need formal evaluation, but hold promise to save financial and human resources.

There is a similar precedent from the World Health Organization for prioritizing household contacts when an index case of tuberculosis is identified [11]. In tuberculosis, household screening has been shown to dramatically improve prevention, early diagnosis, and outcomes [12, 13]. Similar to tuberculosis, RHD has a strong environmental component. RHD originates from group A Streptococcus (GAS), which is endemic in areas of poverty and overcrowding. While the risk of invasive GAS infection among household contacts is only mildly increased when an index case is identified [14], it is likely that family members are exposed to the same streptococcal strains at a similar frequency over time. Thus, while familial chemoprophylaxis is not recommended for individual invasive streptococcal infections [15], our data suggest familial echocardiographic screening, once RHD is identified, may be worthwhile.

In addition to a shared environment, first-degree relatives also share genetic susceptibility, which is thought to play a crucial role in RHD development [2, 16]. Engel et al. reported a meta-analysis of twin-studies that included 435 twin pairs between 1933 and 1964[4]. The pooled concordance risk for ARF was 44% in monozygotic twins and 12% in dizygotic twins (OR 6.39, p<0.001), with an estimated heritability of 60%. To date, targeted genomic investigations have examined select genes involved in immune regulation including human leukocyte antigens (HLA), transforming growth factor-beta1, toll-like receptor 5, angiotensin I-converting enzyme gene, PTPN22, and signal transducers and activators of transcription (STATs) gene polymorphisms [17–23]. The most robust data comes from studies of the major histocompatibility complex human leukocyte antigens [3, 24], with many finding polymorphisms within the HLA-DR locus, including a study from Uganda [25]. However, most of these investigations have been small and no single or combination haplotype has consistently emerged [3, 24]. In this study, we cannot separate the influence of host susceptibility from that of the shared environment; our phenotypic data supports the concept of genetic predisposition in RHD. Future genome-wide association studies are needed to link phenotype to genotype and elucidate the drivers of familial susceptibility.

While not the primary objective of this investigation, our study design captured some of the first data on the community burden of RHD in adults. We found an unanticipated high prevalence of latent RHD among adult females (10/107 or 9.3% of those screened), while no cases were identified in adult males (0/43 screened). These findings are similar to those by Paar et al, who reported a preponderance of female adults both available for screening and having RHD in Nicaragua (91% of all adult cases of definite RHD)[26].

The high prevalence of definite RHD also did not vary between the mothers of cases and those of controls. We hypothesize that these women may have been at particularly high risk due to historical events in the region. Gulu, Uganda, the site of our study, was subject to a major humanitarian crisis between 1996 and 2007, when most of the included mothers would have been children or adolescents. This crisis resulted in families being displaced to refugee camps, where poor sanitation, overcrowding, and constrained access to primary care–all drivers of streptococcal disease–were commonplace. Studies examining the impact of these camps have found a disproportionate impact on woman’s health during this time period [27,28]. While more extensive survey of adult populations in sub-Saharan Africa are needed to confirm, we speculate that the extremely high prevalence of RHD among women in Gulu may not be replicated to this extreme in the wider population. Further echocardiographic studies of latent RHD prevalence in adult populations are needed, in particular as RHD in pregnancy carries high risks of maternal and fetal morbidity and mortality [29,30].

Our study has several limitations. While we were able to capture most mothers and over three-quarters of siblings for screening, we were able to screen less than half of the fathers. This was due to both higher levels of paternal mortality and for those fathers who were alive, high rates of living and working outside of Gulu. Absentee rates for fathers were comparable between cases and controls, but we cannot accurately determine prevalence of latent RHD among fathers. Additionally, a greater number of siblings were captured from RHD positive compared to RHD negative cases. While there was no difference in death rates between siblings and no evidence of poorer cardiovascular health captured among reasons for sibling non-attendance, the impact of this difference cannot be known. We included as RHD positive index cases both children identified with borderline and with definite RHD. While our most significant findings were associated with a sub-analysis of siblings of definite RHD cases, it is possible that the inclusion of borderline cases weakened our overall phenotype, and study power, which could result in underestimation of risk. It is also important to remember that the total number of RHD cases in our families was small, leading to wide confidence intervals and less certainty in our findings, making them more hypothesis-generating than definitive. Finally, we did not specially control for variations in socio-economic status level between families. However, index controls were recruited from the same schools as cases to indirectly equilibrate socio-economic conditions between groups, and the number of people per household did not differ between cases and controls.

It is also necessary to point out that many important practical and logistical questions remain before a public health strategy that includes echocardiographic screening can be broadly recommended. Our study demonstrates that, given the right resources, large-scale echocardiographic screening is feasible in low-income impoverished areas of the world. However, healthcare resources in most endemic areas are highly constrained, and lack of human and financial resources commonplace. Studies examining optimal training strategies and use of less-expensive handheld echocardiography in the hands of non-experts are beginning to address these barriers [31–34]. The natural history of latent RHD, in particular the category of borderline RHD, is unknown. Natural history studies of children with latent RHD are ongoing and may provide answers [32, 35, 36, 37].

### Conclusions

In conclusion, siblings of RHD positive cases with any RHD are more likely to have definite RHD and the relative risk is highest if the index case has definite RHD. Future screening programs should consider implementation of sibling screening following detection of an RHD positive index case. Follow-up of this cohort is needed to determine if latent RHD that exists in more than one family member is more likely to persist and progress. Future studies should prioritize implementation research to answer questions of how RHD screening can best be integrated into existing healthcare structures, ensuring practical and sustainable screening programs.

## Supporting Information

S1 Table World Heart Federation Criteria for the Echocardiographic Diagnosis of Latent Rheumatic Heart Disease (RHD). (DOCX)
