# Supplementary material for: Targeted Echocardiographic Screening for Latent Rheumatic Heart Disease in Northern Uganda: Evaluating Familial Risk Following Identification of an Index Case
Source: PLoS Negl Trop Dis. 2016 Jun 13;10(6):e0004727. doi: 10.1371/journal.pntd.0004727 (PMC4905680; doi:10.1371/journal.pntd.0004727)
Supplement: S1 Table — (DOCX) [file pntd.0004727.s001.docx]

S1 Table: World Heart Federation Criteria for the Echocardiographic Diagnosis of Latent Rheumatic Heart Disease (RHD).

| **Patients ≤ 20 years of age** | |
| --- | --- |
| **Definite**: Either A, B, C, or D | |
| 1. Pathologic mitral regurgitation and at least two morphologic features of RHD of the mitral valve. | |
| 1. Mitral stenosis with mean gradient ≥4 mmHg* | |
| 1. Pathologic aortic regurgitation and at least two morphologic features of RHD of the aortic valve | |
| 1. Borderline disease of both the aortic and mitral valves*** | |
| **Borderline**: Either A, B, or C | |
| 1. At least two morphologic features of RHD of the mitral valve | |
| 1. Pathologic mitral regurgitation | |
| 1. Pathologic aortic regurgitation | |
| **Patients > 20 years of age** | |
| **Definite**: Either A, B, C, or D | |
| 1. Pathologic MR and at least two morphologic features of RHD of the mitral valve. | |
| 1. Mitral stenosis with mean gradient ≥4 mmHg* | |
| 1. Pathologic aortic regurgitation and at least two morphologic features of RHD of the aortic valve, only in individuals <35 years** | |
| 1. Pathological aortic regurgitation and at least two morphological features of RHD of the mitral valve | |
| **Pathologic Mitral Regurgitation**  (all criteria must be met) | **Pathologic Aortic Regurgitation**  (all criteria must be met) |
| Seen in two views | Seen in two views |
| Jet length ≥2 cm (in at least one view) | Jet length ≥1 cm (in at least one view) |
| Velocity ≥3 m/s for one complete envelope | Velocity ≥3 m/s for one complete envelope |
| Pansystolic jet in at least one envelope | Pandiastolic jet in at least one envelope |
| **Morphologic features of the mitral valve** | **Morphologic features of the aortic valve** |
| Anterior leaflet thickening ≥3 mm | Irregular or focal thickening |
| Chordal thickening | Coaptation defect |
| Restricted leaflet motion | Restricted leaflet motion |
| Excessive leaflet tip motion during systole | Prolapse |
| *Congenital mitral valve anomalies must be excluded. Furthermore, inflow obstruction due to non-rheumatic mitral annular calcification must be excluded in adults; ** Bicuspid aortic valve, dilated aortic root, and hypertension must be excluded. ***Combined aortic regurgitation and mitral regurgitation in high prevalence regions and in the absence of congenital heart disease is regarded as rheumatic. | |
